# Effect of Vowel Auditory Training on the Speech-In-Noise Perception among Older Adults with Normal Hearing

**DOI:** 10.22038/ijorl.2019.33433.2110

**Published:** 2020-07

**Authors:** Atta Heidari, Abdollah Moossavi, Fariba Yadegari, Enayatollah Bakhshi, Mohsen Ahadi

**Affiliations:** 1 *Department of Audiology, Faculty of Rehabilitation, Hamadan University of Medical Sciences, Hamadan, Iran.*; 2 *Department of Otolaryngology and Head and Neck Surgery, School of Medicine, Iran University of Medical Sciences, Tehran, Iran.*; 3 *Department of Speech Therapy, University of Social Welfare and Rehabilitation Sciences, Tehran, Iran.*; 4 *Department of Biostatistics, University of Social Welfare and Rehabilitation Sciences, Tehran, Iran.*; 5 *Department of Audiology, Rehabilitation Research Center, School of Rehabilitation Sciences, Iran University of Medical Sciences, Tehran, Iran.*

**Keywords:** Aging, Fundamental Frequency (F0), Speech-in-Noise Perception, Vowel Auditory Training

## Abstract

**Introduction:**

Aging reduces the ability to understand speech in noise. Hearing rehabilitation is one of the ways to help older people communicate effectively. This study aimed to investigate the effect of vowel auditory training on the improvement of speech-in-noise (SIN) perception among elderly listeners.

**Materials and Methods:**

This study was conducted on 36 elderly listeners (17 males and 15 females) with the mean±SD of 67.6±6.33. They had the normal peripheral auditory ability but had difficulties in SIN perception. The samples were randomly divided into two groups of intervention and control. The intervention group underwent vowel auditory training; however, the control group received no training.

**Results:**

After vowel auditory training, the intervention group showed significant changes in the results of the SIN test at two signal-to-noise ratios of 0 and -10 and the Iranian version of the Speech, Spatial, and Qualities of Hearing Scale, compared to the control group (P<0.001). Regarding the Speech Auditory Brainstem Response test, the F_0_ magnitude was higher in the intervention group (8.42±2.26), compared to the control group (6.68±1.87) (P<0.011).

**Conclusion:**

This study investigated the effect of vowel auditory training on the improvement of SIN perception which could be probably due to better F_0_ encoding and receiving. This ability enhancement resulted in the easier perception of speech and its more proper separation from background noise which in turn enhanced the ability of the old people to follow the speech of a specific person and track the discussion.

## Introduction

The mixture of sounds reaching from different sources and directions to the ears is normally separated into their original structures by the unique ability of the auditory system (1). In this process, the central auditory nervous system reduces the power of the interrupting noises and augments the main signal (2-7). Some people with normal hearing thresholds face the problems of understanding normal speech in noisy conditions. This problem generally categorized as the (central) auditory processing disorder (cAPD) has been primarily described for a group of learning deficient children; however, nowadays, it is attracting too much attention for diagnosis and possible management in elderlies since some older adults also complain of the same problem which does not need amplification due to their normal or near-normal hearing thresholds (2,8-11). These people suffer from personal and social difficulties (i.e., listening to radio broadcasting, watching TV programs, and mutual communications) leading to their isolation from social life (10,12-15). The trend of population aging across the world and in our country urges us to find a solution for cAPD in elderlies. Environmental sounds can be generated by different sound sources. In complex acoustic environments, various sound sources are simultaneously active and a combination of their frequency spectra will reach the audience. The auditory system can segregate and group the different components of the incoming mixture of auditory information based on their characteristics, thereby identifying any specific stream from others (1). Bregman (1990) described this process for the first time and named it Auditory Scene Analysis (ASA) (2,3). Moreover, Bregman believes that the segregation process is an automatic or primitive phenomenon and operates before starting the attention or top-down control. Numerous evidence has demonstrated that the bottom-up sensory processes which are the sources of sound segregation, act in pre-attentive (bottom-up) stages, and indicate the segregation of auditory streams are performed before the selection of any specific stimuli (4-9). The most important result of this automatic segregation of mixture of incoming auditory stimuli from different sources is the easier selection of special stream of desired speech from any type of noise and better processing and understanding (8).

The process of simultaneous sound streams has been segregated from different sources according to their frequency contents and harmonic relations which results in the separation of sound streams from any specific external source. This leads to an independent perception of each stream which is finally shaped the same as a specific representation in the central auditory system (2,3).

Speech is an acoustic signal including fast variations in spectral and temporal features and is composed of acoustic segments and features (segmental and supra-segmental). Any of these segments play an important role in the formation of correct speech perception (10,11). From the acoustical point of view, speech is composed of vowels and consonants which comprise the segmental parts of the speech. Vowels present the stable parts of speech (i.e. fundamental frequency, first and second formants). The data from these formant features include phonetic and prosodic information which along with consonants forms the basis for speech perception (12). 

The segregation of different sounds in the auditory system happens at least in two essential patterns according to the temporal and frequency structure of the incoming sounds. The crucial importance of the fundamental frequency (F_0_) and its earlier low-frequency harmonics of complex stimuli, such as speech has been shown in the detection of their pitch and perceptual segregation of them (3,11,12). It has been revealed that subcortical encoding of the F_0_ is an important factor in the perception of speech in noisy conditions (13) which is most probably encoded in the upper part of the brain stem, especially in the inferior colliculus (14). From the ASA point of view, vowels are the available tools for the discrimination of the simultaneous sounds for correct speech perception. As quasi-periodic sounds, vowels facilitate the understanding of sound pitch by the human auditory system (11,12). It seems that the first step in speech perception is the extraction and identification of F_0_, pitch, properties of formants, vowels, and their harmonic relationships correctly and automatically, especially in noisy conditions. (11,13,15-18). Therefore, hearing the vowels will result in better speech discrimination in different situations, especially in environments crowded with competitive sounds and speech.

Previous studies have shown a reduction in perceiving F_0_ and coding ability in these people. It has also been proven that the ability of older people in speech-in-noise (SIN) perception will also decline due to this reduction and the resultant deficit in pitch perception (10,13,18-21). Anderson et al. (2011) showed that receiving and coding the magnitude of F_0_ is lower in older adults with difficulties in speech perception in a noisy environment (13). 

There is no medical management for cAPD, and auditory training and rehabilitation of the hearing system is one of the main methods to improve the SIN perception due to plasticity in the central hearing system (22-26). The plasticity has been surveyed in children and young adults. The effect of vowel auditory training on the SIN perception has been investigated by investigating the ability of concurrent speech segregation in hearing impaired children (8). Additionally, the impact of music as a periodic sound on the improvement of SIN perception was also proven in children and young people (24-26). It is becoming evident that plasticity can also occur in auditory nervous system of older adults with cAPD. Given the good results for a successful auditory rehabilitation in elderlies (27,28), auditory rehabilitation can be regarded as a potential management for this problem. 

This study aimed to investigate the effect of vowel auditory training (which is dependent on the identification of F_0_ and subsequent formants) on the improvement of the SIN perception among older adults who had difficulties in speech perception in noisy conditions.

## Materials and Methods

In total, 32 adults (aged 60 years or over) with the mean age of 68.9±6.33 years participated in this study which was conducted in Rofaideh Hospital, Tehran, Iran. It should be noted that the majority of the participants were male (n=17), and they were recruited from Yas senior nursing home and health houses of Tehran Municipality, Tehran, Iran. Subsequently, they received a complete explanation about research objectives and procedures, and written informed consent was obtained from them. The inclusion criteria were: 1) right-handedness (confirmed by Persian version of Edinburgh Handedness Inventory questionnaire), 2) monolingual (Persian language as their mother language), 3) normal external auditory canals and intact tympanic membranes, and 4) acceptable hearing thresholds without any history of ear diseases, head trauma or accident, brain surgeries, epilepsy, and nervous system medication use. The mean hearing thresholds at the range of 250-4000 Hz (octave band) for the subjects were equal or better than 25 dB HL in both ears. Moreover, the threshold of each of four frequencies equal or better than 40 dB HL with a maximum mean difference of the threshold for each similar frequency in both ears did not exceed 5 dB HL.

Normal tympanometry and acoustic reflex, as the sign of normal performance of the tympanic membrane and middle ear of the participants, were the prerequisites. Mini-Mental State Examination was utilized to screen the normality of the cognitive function of the subjects. In the next stage, the participants were randomly divided into two groups of intervention (8 males and 8 females) and control (9 males and 7 females). The mean±SD ages of the intervention and control groups were 67.56±5.68 and 70.25±6.84, respectively. Following that, SIN perception tests were carried out using the Persian version of temporal resolution test in adults, including four standardized 50-word lists with continuous noise in two signal-to-noise ratios of 0 and -10 (29,30). Furthermore, the participants were asked to complete the Iranian version of Speech, Spatial, and Qualities of Hearing Scale (SSQ) questionnaire. This questionnaire translated from the original version of the SSQ questionnaire with confirmed reliability and validity is one of the most important self-evaluating tools in the field of communicative disorders due to hearing loss, especially among old people (31). This scale consists of 47 statements classified in speech perception, spatial hearing, and hearing quality subgroups. The participants evaluated their abilities in any of these statements using a 10-degree horizontal scale in which 0 and 10 indicate the minimum and maximum abilities, respectively. 

Furthermore, each participant underwent Speech Auditory Brainstem Response (ABR) test by Bio-Logic Navigator Pro system with Cz for the noninverting electrode and right earlobe for inverting one. The earth electrode was conventionally placed on the forehead. The impedance of all electrodes was kept below 5 kΩ with a maximum difference of 1.5 kΩ. The insert earphone ER-3A (Etymotic Research, Elk Grove Village IL) was used to deliver the standard stimulus of the BioMARK module and the synthesized stop consonant /da/ with a duration of 40 ms. The mentioned stimulus has an initial noise burst, a formant transition between the consonants, and a later steady-state vowel containing the F_0_ rising linearly from 103 to 125 Hz. 

The voicing begins after 5 ms containing the onset release burst during the first 10 ms. The frequency content of the stimulus shows that the first formant (F_1_) rises linearly from 220 to 720 Hz, and for the second formant (F_2_), it decreases from 1700 to 1240 Hz. The third formant (F_3_) has a slight fall from 2580 to 2500 Hz, whereas the fourth (F_4_) and fifth (F_5_) formants remain changeless at 3600 and 4500 Hz, respectively. The stimulus costume option in Biologic AEP software (version 7.0) was used to deliver the stimulus in alternating polarity mode and presentation rate of 10.9 per second. The intensity of the stimulus was fixed at 80 dB SPL and calibrated by 2-cm3 DB-0138 coupler Bruel and Kjaer Type 2203 audiometer and a microphone with a one-inch diameter. The filter setting was 100-2000 Hz with a sampling rate of 1024, and a time window equal to 85.33 ms (containing a 15 ms pre-stimulus time) was also employed in this study. According to the current standards, all stimuli were delivered to the right ears. Artifact rejection was set, and traces exceeding ±23.8 mV were rejected from the average. In total, two sub-averages of 2000 sweeps making a total of 4000 artifact-free responses were obtained. 

The environmental condition of the participants included calm conditions and reclining position on a comfortable chair with closed eyes in an acoustic room enjoying low light and low electrical noise. The participants had no cognitive task during the test. The Mat Lab software (version R2013a, The Math Works, Inc., Natick, Massachusetts, USA) was used for spectral analysis of the obtained responses.

After the tests, vowel auditory training was performed for the intervention group. Auditory training involved training 6 vowels of /æ/, /e/, /a/, /i/, /o/, and /u/ in the form of nonsense single syllables (8) for 5 weeks (32). For instance, syllables, such as /pæ /, /ʃæ /, /sæ /, /hæ/, and /kæ / were presented by a male pronouncer in a calm and echo-less space from a 1-m distance behind the participant in a most comfortable level. Subsequently, the participants had to identify and express them. This trend was repeated for the other vowels (i.e, /e/, /a/, /i/, /o/, and /u) (8). 

The 1-hour auditory training sessions were held 3 times a week for 5 weeks (32) for all the mentioned vowels. During these sessions, the states of answering to the items were recorded, and if the participants made a mistake in answering any case, regardless of their errors, the next item would be pronounced. In this way, all the items would be presented and exercised randomly and equally during each session.

After completing the rehabilitation sessions, the SIN test, the Iranian version of the SSQ questionnaire, and Speech ABR tests were administered again, and the results of the test were recorded and compared after 5 weeks with those of the control group.

The data were analyzed in SPSS software (version 16). Moreover, the Shapiro-Wilk test was used to assess the normality of data. Regarding the data normality, t-test and covariance analysis were employed to analyze the data obtained from both groups before and after the intervention. Moreover, the Pearson correlation coefficient was utilized to calculate the correlation of the results of the behavioral tests with an F_0_ range of variations.

## Results

Table 1 tabulates the results of the SIN test at the presence of noise in two signal-to-noise ratios of 0 and -10 for the control and intervention group after the intervention. Before the intervention, the mean values of SIN perception were 50 and 32 in two signal-to-noise ratios of 0 and -10 in the intervention group, respectively.

On the other hand, these corresponding values were 53.12 and 24.87 for the control group in the already-mentioned order. The scores of the intervention group were significantly higher than those before the intervention and higher than those in the control group after the intervention (P<0.001).

**Table 1:** scores of speech-in-noise perception test before and after training for both groups

**Signal to noise ratio**	**Group**	**Before**		**After**	
		**Mean**	**SD**	**Mean**	**SD**
SNR 0	test	50	5.16	58.25	6.76
	control	53.12	8.06	52.87	7.99
SNR -10	test	23	5.79	31.12	7.99
	control	24.87	5.58	25.25	6.68

Table 2 also lists the results of the Iranian version of SSQ in three subscales of speech perception, spatial hearing, and hearing quality for both groups before and after the intervention. Before the intervention, the mean SSQ total scores were 7.12 and 7.04 for the intervention and control groups, respectively. Moreover, the intervention group obtained significantly higher scores after the training, compared to the control group (P<0.001).

**Table 2:** score mean of the Iranian version of SSQ questionnaire before and after training for both groups

**Item**	**Group**	**Before**		**After**	
		**Mean**	**SD**	**Mean**	**SD**
speech perception	test	7.12	.24	7.66	.29
	control	7.1	.4	7.08	.38
spatial hearing	test	7.07	.23	7.56	.32
	control	7.01	.55	7.01	.52
hearing quality	test	7.18	.35	7.52	.32
	control	7.02	.59	7.05	.45
Total score	test	7.12	.19	7.58	.2
	control	7.04	.46	7.05	.37

Spectral analysis of measurement accuracy and the level of neuron phase locking in F_0_, first formant F1, and higher formants HF were 

employed, and their receiving and encoding in the brainstem were obtained in this study(Table.3). 

**Table 3:** mean scores of Speech ABR response spectral analysis before and after training in both groups

**Spectral magnitudes (mV)**	**Group**	**Before**		**After**	
		**Mean**	**SD**	**Mean**	**SD**
F0	test	6.55	2.7	8.42	2.26
	control	7.4	3.06	6.68	1.87
F1	test	1.07	.22	1.18	.33
	control	1.09	.29	1.05	.32
HF	test	.48	.11	.58	.2
	control	.5	.2	.48	.15

Based on the spectral analysis, F_0_ receiving and encoding values were 8.42 (SD±2.26) and 6.55 (SD±2.7) in the intervention group after and before the intervention, respectively. Before the intervention, the F_0_ receiving and encoding values were 6.55 and 7.4 for the intervention and control groups, respectively. Moreover, after the intervention, the F_0_ receiving and encoding values of the intervention group were significantly higher than those in the control group (6.68 with SD±1.87) (P<0.001). After the intervention, the results of the SIN test showed a high correlation of F_0_ magnitude with the signal-to-noise ratios of 0 (r=0.35, P<0.047) and -10 (r=0.37, P<0.037), as well as the SSQ score (1=0.61, P<0.001). 

## Discussion

This study included 32 elderly listeners with normal hearing ability but difficulties in SIN perception. The subjects participated in 15 sessions of vowel auditory training, and the Iranian version of the SSQ questionnaire, SIN perception, and speech ABR tests were used before and after the intervention.

The results of the SIN perception test before the intervention were in good agreement with the findings of a study conducted by Stuart et al. and Jafari et al.; however, they were not in line with the results of a study performed by Omidvar et al. on young people with normal hearing (the scores were lower) (30,33,34). Previous studies have also revealed a decrease in the SIN scores of the elderly listeners which could be attributed to a decrease in the ability to discriminate simultaneous sounds and receiving the target speech among them. Moreover, the scores of the SSQ questionnaire before the intervention were consistent with the results of a study carried out by Singh et al. who obtained a score of 7.7. However, in comparison with the normal hearing ability of the young people, these scores show a decrease which could be due to the impact of aging on the reduction of communicative abilities as the results of the decline in the ability to segregate simultaneous sounds (35). In the same line, the results of speech ABR before the intervention coincide with the findings of a study conducted by Anderson et al. and Vongpaisal et al. (13, 36). Nonetheless, they showed a decrease, compared to the results of the studies performed by Ahadi et al (2014) and Heidari et al (2018) on young people with a normal hearing ability (21,37). One of the reasons could be a decrease in neuron phase-locking and temporal resolution in subcortical neuron response due to a decrease in the gamma-aminobutyric acid-ergic inhibition since F_0_ receiving and encoding are performed by the neuron phase locking of the brainstem (38,39). Subcortical age-related changes in F_0_ encoding could be one of the reasons for weak speech perception in noisy environments in older adults because lower ability in receiving F_0_ will cause more problems in SIN perception. It is thought that older people have perceptual deficits due to difficulties in auditory scene analysis, such as perceiving speech in noisy conditions and segregation of acoustic information into multiple streams. When an elderly complains of such problems without measurable hearing loss, it can be concluded that some central auditory mechanisms may be the basis of the problem.

After vowel auditory training, the intervention group members showed a significant improvement in SIN scores, responses to SSQ items, and F_0_ receiving and encoding which was not observed in the control group. Since this study only used vowel auditory training, this improvement could be attributed to this condition. Numerous studies have shown that brainstem plasticity is dynamic, and some variations may occur in receiving areas based on acoustic experiences, stimuli, and following auditory training (23-25,32). The impact of vowel auditory training on the improvement of speech segregation has also been proven in hard-of-hearing children (8). It has been shown that vowel auditory training has a remarkable benefit in complex listening conditions requiring listeners to parse a complex auditory scene with multiple sound objects. In the current study, after short-term vowel rehabilitation by nonsense syllables which resulted in an improvement in F_0_ receiving, it can be concluded that some degrees of plasticity have occurred in the brainstem of old adults. This finding is consistent with the results of a study conducted by Song et al. (2008) who showed the impact of auditory training on the plasticity of brainstem and improvement of F_0_ receiving among adults (40). Regarding the findings of this study showing weak F_0_ receiving and encoding before training, it can be concluded that elderlies with even normal hearing thresholds may have deficits in speech processing and perception at the presence of noise. Improvement in the mentioned items after the training indicates that this deficit can be compensated even in old ages due to the possibility of brainstem plasticity.

The periodic vibration of the vocal folds results in a low-frequency component (i.e. the F_0_ of speech, contributing to the pitch of an individual’s voice). Prosodic aspects of the speech are reflected by F_0_, and its encoding is the single important factor for identifying the speaker (41,42). The F_0_ is a key tool in the perception of speech pitch which is based on the ASA insight and is involved in the segregation of the sound source (10,13,42). The results of the studies conducted by Anderson et al. showed the effect of better F_0_ receiving on the segregation of the concurrent vowels (13, 43). Therefore, it can be expected that proper training can improve the SIN perception by increasing F_0_ receiving and encoding in the brainstem. Our findings confirm these facts since the behavioral and neurophysiological representation of the pitch showed improvements in our subjects after rehabilitation which reflect the enhancement of synchronization of neuronal firing to the stimulus F_0_. This enhancement may be either due to an additional group of neuron start to fire at the rate of the stimulus F_0_, or better synchronization firing by the same population of neurons, or a combination of both. This can prevent many of the communicational problems of the elderly people which occur due to their aging leading to their isolation. The strong correlation between the behavioral test results and F_0_ magnitude also confirmed this theory. 

## Conclusion

Figure 1 shows a great mean of speech ABR responses in the frequency domains of the two groups. Our findings revealed that the elderly with normal hearing sensitivity and a SIN difficulty could benefit from the vowel auditory training program. This training improved F_0_ receiving and encoding as well as speech in noise perception. According to the current study, it seems that the plasticity of ASA is still maintained in old age. This study may indicate potential or future training programs for the elderly to help them overcome speech in noise difficulties. In case of obtaining positive results in more extensive studies, SIN perception and vowel separation ability can be recommended as a general protocol for investigating the SIN perception problems of the old adults.
